# Differential Effects of Activated Human Renal Epithelial Cells on T-Cell Migration

**DOI:** 10.1371/journal.pone.0064916

**Published:** 2013-05-22

**Authors:** Martijn W. H. J. Demmers, Carla C. Baan, Els van Beelen, Jan N. M. IJzermans, Willem Weimar, Ajda T. Rowshani

**Affiliations:** 1 Department of Internal Medicine, Division of Nephrology and Transplantation, Erasmus MC – University Medical Center Rotterdam, Rotterdam, the Netherlands; 2 Department of Immunohematology and Blood Transfusion, Leiden University Medical Center, Leiden, the Netherlands; 3 Department of Surgery, Division of Transplant Surgery, Erasmus MC – University Medical Center Rotterdam, Rotterdam, the Netherlands; Istituto Superiore di Sanità, Italy

## Abstract

**Background:**

Renal tubular epithelial cells (TECs) are one of the main targets of inflammatory insults during interstitial nephritis and kidney transplant rejection. While Th1 cells are know to be essential in the pathogenesis of rejection, the role of Th17 is still under debate. We hypothesize that TECs modulate the outcome of rejection process by production of distinct chemokines and cytokines that determine the attraction of different T-cell subsets. Therefore, we studied differential effects of activated human renal epithelial cells on T-cell migration.

**Methods:**

Human primary TECs were stimulated by IFN-γ and TNF-α *in vitro*. Chemokines and cytokines produced by activated TECs were measured using Luminex or ELISA. Chemotaxis assay was performed using activated peripheral blood mononuclear cells composed of CD4^+^CXCR3^+^ and CD4^+^CCR6^+^ T cells migrating towards stimulated and unstimulated TECs.

**Results:**

While activated TECs secreted abundant amounts of the pro-inflammatory cytokines IL-6 and IL-8, the T helper cell differentiation cytokines IL-1β, IL-12p70, IL-23 or TGF-β1 were not produced. The production of Th1 chemokines CXCL9, CXCL10 and CCL5 were significantly upregulated after TEC stimulation. In contrast, Th17 chemokine CCL20 could not be detected. Finally, activated TECs attracted significantly higher numbers of CD4^+^CXCR3^+^ T cells as compared to unstimulated TECs. No migration of CD4^+^CCR6^+^ T cells could be observed.

**Conclusion:**

Activated primary renal tubular epithelial cells do not attract Th17 cells nor produce cytokines promoting Th17 cell differentiation in our experimental system mimicking the proinflammatory microenvironment of rejection.

## Introduction

Tubular Epithelial Cells (TECs) comprise more than 75% of renal parenchymal cells. Their susceptibility and resistance to both inflammation and apoptosis directs the long-term function of kidney transplants, as tubular injury can be a major cause of nephron loss [1]. As such, tubulitis is the diagnostic hallmark of acute cellular rejection and can potentially lead to irreversible structural graft damage.

To date, important progress has been made in understanding the pathogenesis of rejection. So far, mechanisms of rejection entail a multi-cellular inflammatory process where local environment and multi-directional interplays between T cells and parenchymal cells such as tubular epithelial cells will determine the final outcome [2], [3].

Classically, the proinflammatory Th1 cell is thought to represent one of the dominant immune cell types involved in induction and maintenance of acute cellular rejection [4], [5]. On the contrary, the role of Th17 cells in the rejection process is not clear yet [6]. Th1 cells express IFN-γ and the transcription factor T-bet [7]. Th17 cells are defined by the expression of the cytokine IL-17 and the retinoic acid-related orphan receptor γ (RORγ) [8]. Distinct cytokines drive the differentiation of naïve CD4^+^ T cells towards different T cell subsets. IL-12 and IFN-γ are considered to be the main T helper 1 associated cytokines and IL-1β, IL-6, IL-23 and TGF-β1 are T helper 17 associated counterparts [4].

At present, controversial data exist with regard to the involvement and the role of Th17 cells during kidney transplant rejection [6]. Despite some experimental murine data directly linking Th17 cells to rejection, most studies in clinical transplantation are limited to the detection of IL-17. IL-17 has been detected by immunofluorescent staining in biopsies from acutely rejecting transplants and not in pretransplant biopsy samples or healthy kidneys [9]. Increased IL-17 mRNA and protein expression levels in kidney biopsies and urine samples from patients with subclinical rejection underline the involvement of IL-17 in the process of rejection [10]. Yapici et al. reported that the majority of IL-17 producing cells in kidney transplant biopsies undergoing acute rejection are mast cells or neutrophils and not T-lymphocytes [11]. These data suggest that IL-17 may play a role during the rejection process.

Th1 cells preferentially express the chemokine receptors CCR5 and CXCR3 migrating towards CCL5 and CXCL10 which are expressed by TECs and present during kidney transplant rejection [12]–[16]. Th17 cells are characterized by the simultaneous expression of IL-17 and the chemokine receptor CCR6 directing the most robust chemotaxis towards CCL20 [17], [18]. In a prospective biopsy-controlled study it was found that local CCL5 and CXCL10 produced by TECs led to the directional movement of activated CXCR3 and CCR5 bearing T cells into the kidney transplant mediating acute rejection [14]. Accordingly, CCL20 expression was shown by immunohistochemical staining of both infiltrating leucocytes and tubular epithelial cells in kidney biopsies obtained from patients experiencing rejection [19].

We hypothesize that TECs modulate the outcome of the inflammatory process by the production of distinct chemokines and cytokines that determine the attraction, activation and further differentiation of T-cell subsets. In the present study, we specifically aim to investigate whether TECs after stimulation by IFN-γ and TNF-α, which are instrumental inflammatory cytokines during rejection, have the potential to attract Th1 and Th17 T-cell subsets. Chemokine and cytokine production by IFN-γ/TNF-α activated primary human TECs as well as their functional potential to attract CD4^+^ T cell subsets were studied.

## Materials and Methods

### Culture of primary tubular epithelial cells

Primary tubular epithelial cells were cultured from cortical tissue of human kidneys obtained at the time of transplantation as previously described [20], [21]. TECs were cultured in serum-free Dulbecco's modified Eagle's medium and Ham F12 (BioWhittaker, Verviers, Belgium) in a 1∶1 ratio, supplemented with insulin (5 µg/ml), transferrin (5 µg/ml), selenium (5 ng/ml), hydrocortisone (36 ng/ml), tri-iodothyronine (40 pg/ml) and epidermal growth factor (10 ng/ml) (all from Sigma). Cell growth was maintained in a T75 cell culture flask (Greiner Bio-One, Essen, Germany). For passages of cell cultures, cells were washed twice with phosphate buffered saline (PBS) and trypsinized with 0.05% trypsin-EDTA at 37°C and then washed in Dulbecco's modified Eagle's medium and Ham F12 in a 1∶1 ratio supplemented with 10% heat inactivated FBS. Cells were used between passages 2 and 6 of culture. The specific outgrowth of TEC was confirmed by morphologic appearance and immunofluorescence staining (CD13^+^, CD26^+^ and CD90^-^) (data not shown).

### TEC activation experiments

TECs were seeded in 24 well plates at a density of 2×10^5^ TEC/ml in the above described medium. After one day medium was replaced with TEC culture medium with or without addition of cytokines. TECs were stimulated with 50 ng/ml human recombinant IFN-γ (U-Cytech, Utrecht, the Netherlands) and/or 20 ng/ml human recombinant TNF-α (PeproTech, London, UK) for 24 h, 48 h and 72 h. Supernatants were harvested and stored at −80°C until analysis.

### Flow cytometry

TECs were harvested via trypsinization after 24, 48 and 72 hours. TECs washed with cold PBS supplemented with 0.5% albumin (PBSA 0.5%) and stained for CD13 PE-Cy7, CD26 FITC, CD86 FITC, HLA-I APC, HLA-DR APC-Cy7 (all BD Biosciences), CD40 PerCP-Cy5.5 (Biolegend), CD80 PE (Serotec), CD90 APC (R&D systems) at 4°C for 30 minutes. Cells were washed twice with cold PBSA 0.5% following flow cytometric analysis. Fifty thousand events were acquired from each tube by a FACSCanto II flow cytometer (BD Biosciences, San Jose, CA). Data was analyzed with FACS Diva 6.1 software (BD Bioscience).

### Quantitative real time PCR

Total RNA was isolated using the High Pure RNA Isolation kit (Roche Applied Science, Almere, The Netherlands), according to the manufacturer's instructions. RNA concentrations were measured by using the Nanadrop ND-8000 Spectrophotometer (Isogen Life Science, De Meern, the Netherlands). First-strand complementary DNA (cDNA) reaction was performed from 500 ng of the isolated RNA. A quantitative RT-PCR was used to quantify the amount of MIP-3α in the samples. Assay-on-demand products for the detection and quantification of MIP-3α (hs00171125_m1) mRNAs were designed by Applied Biosystems (Foster City, CA). A 5 µL sample of cDNA was added to 20 µL PCR mixture containing 12.5 µL Universal PCR Master Mix (Applied Biosystems), 0.625 µL of each specific primer and probe assay-on-demand mix, and 6.875 µL of water. The RT-PCR reaction was performed with the StepONEplus (Applied Biosystems). The amount of each target molecule was quantified by measuring the threshold cycles (C_t_) on a TaqMan Real-Time PCR system (Applied Biosystems) and was transformed to the number of cDNA copies [2^(40-Ct)^]. The absolute value of the number of MIP-3α mRNA copies was log transformed.

### Cytokine analysis

Production of cytokines by unstimulated and 24 h, 48 h and 72 h IFN-γ/TNF-α stimulated TECs were measured in supernatant using a Bio-Plex multiplex assay (Bio-Rad Laboraties, Veenendaal, the Netherlands) for IL-1β, IL-6, IL-12p70, IL-17, CXLC9 (MIG), CXLC10 (IP-10), CCL5 (RANTES), IL-8 and CCL2 (MCP-1). Samples were analyzed using a Bio-Plex Array Reader with Bio-Plex software. IL-23 (U-CyTech, Biosciences, Netherlands), TGF-β1 (eBioscience, San Diego, USA) and CCL20 (MIP-3α) (R&D Systems, Minneapolis, MN) were measured by ELISA. All immunoassays were performed according manufacturers guidelines.

### Chemotaxis assay

Migration assays were performed using 3 µm pore membrane inserts (ThinCerts; Greiner Bio-One). For each experiment, TECs were seeded in a 24 well plate at a density of 5×10^3^ cells (an optimized cell density; data not shown). Stimulation was performed with 50 ng/ml IFN-γ and 20 ng/ml TNF-α for 24 hours. PBMCs were activated to induce expression of the chemokine receptors CXCR3 and CCR6. PBMCs were stimulated with soluble anti-CD3 (1 µg/ml), anti-CD28 (1 µg/ml) and polyclonal goat anti-mouse Ig (1 µg/ml) (BD Pharmingen), IFN-γ (50 ng/ml, U-Cytech) and IL-2 (200 IU/ml Chiron BV, Amsterdam, the Netherlands) for 3 days. Anti-CD3/CD28 activated PBMCs were washed with medium without serum three times. After 4 hours of incubation at 37°C, migrated CD4^+^ T cells were analyzed for CXCR3 and CCR6 expression by flow cytometry (BD FACS CantoII). Total cell numbers were determined by flow cytometric analysis, performed with a fixed time point and a fixed volume for standardization.

### Ethical statement

This study was approved by the local medical ethical committee of the Erasmus MC - University Medical Center Rotterdam. All patients included in this study gave written informed consent. This study was conducted according to the principles of the Declaration of Helsinki.

### Statistics

Results are expressed as mean ± SEM unless stated otherwise. Cytokines and chemokines present in supernatant were analyzed for statistical significance with GraphPad Prism 5.01 software (Graphpad Software, La Jolla, CA) using non-parametric Friedman test followed by Dunn's post hoc test. Chemotaxis experiments were analyzed with the non-parametric Wilcoxon matched-pairs signed-rank test. *P* values of less than 0.05 were considered statistically significant.

## Results

### IFN-γ and TNF-α stimulation upregulates expression of CD40, HLA-I and HLA-DR by TECs

**Figure 1:**
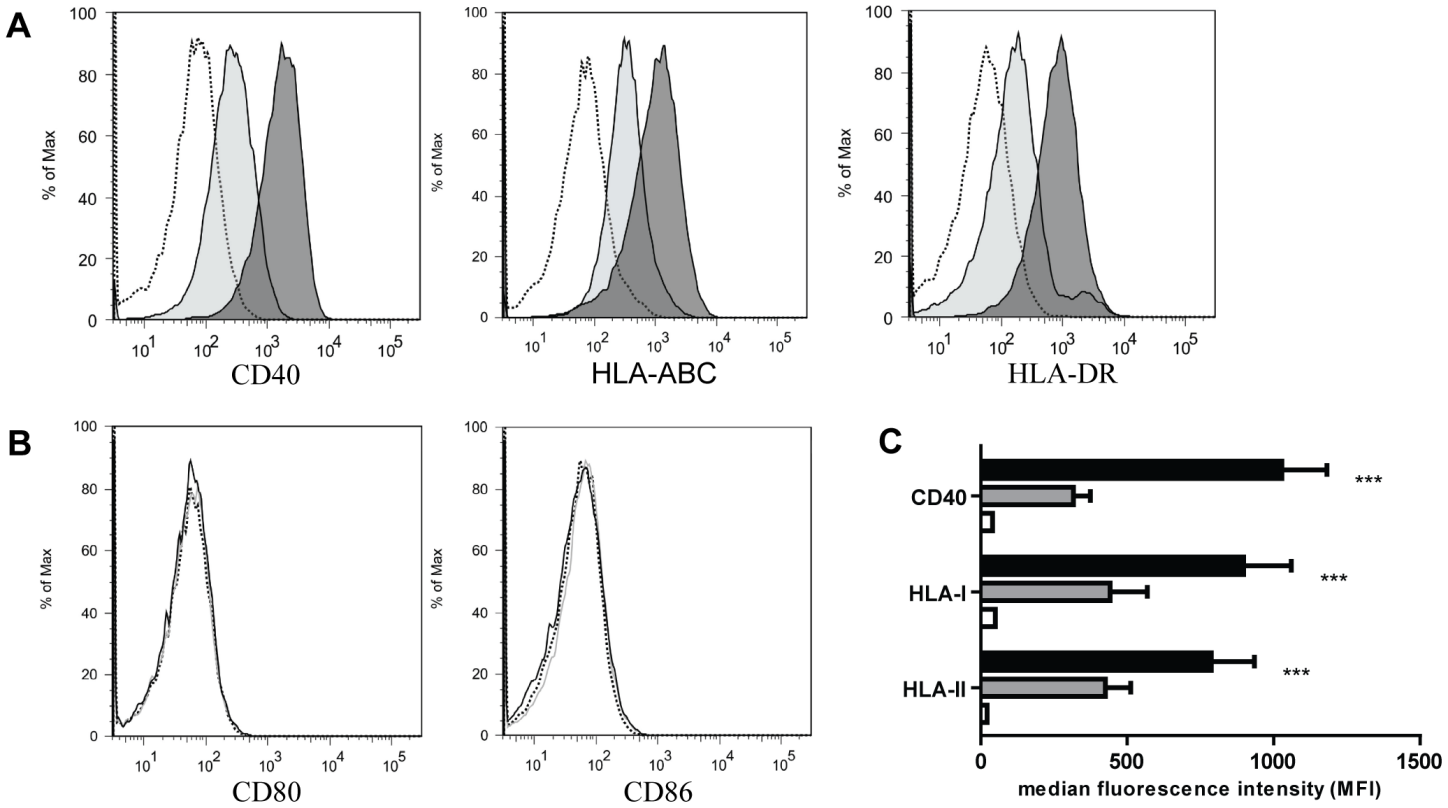
IFN-γ and TNF-α stimulation upregulates expression of activation markers CD40, HLA-I and HLA-DR by TECs. Primary TECs were stimulated with IFN-γ (50 ng/ml)/TNF-α (20 ng/ml) for 24, 48 and 72 hours. (A) Surface staining was performed for the activation markers CD40, HLA-I and HLA-DR. One representative out of 10 experiments is shown. The upregulation of activation markers was observed after 24 h stimulation and still detectable after 72 h stimulation (data not shown). (B) Primary TECs were also stained for the costimulatory molecules CD80 and CD86. The dotted line represents unstained control, grey bar/line represents unstimulated situation and the dark bar/line represents IFN-γ/TNF-α stimulation (C) Quantification of flow cytometric results (mean ± SEM, n = 10) indicating the median fluorescence intensity of the activation markers CD40, HLA-I and HLA-II (n = 3). White bars represent unstained control, grey bars represent unstimulated condition and black bars represent IFN-γ/TNF-α stimulation *** Significant increase (P <0.001).

TECs were cultured with medium alone and in the presence of IFN-γ/TNF-α. After 24 hours of stimulation, CD40, HLA-I and HLA-DR cell surface markers were upregulated by TECs and remained highly present for at least 72 hours as shown by a significantly higher mean fluorescence intensity (MFI) indicating that TECs are activated in this experimental system (P<0.001) (Figure 1A and Figure 1C). Additionally, we investigated the expression of some other TEC related (activation) markers. In line with earlier findings the co-stimulatory molecules CD80 and CD86 were not expressed by TECs irrespective of their stimulation state (Figure 1B).

### TECs fail to secrete Th1 and/or Th17 differentiation cytokines after IFN-γ and TNF-α stimulation while producing abundant amounts of proinflammatory cytokines IL6 and IL8

**Figure 2:**
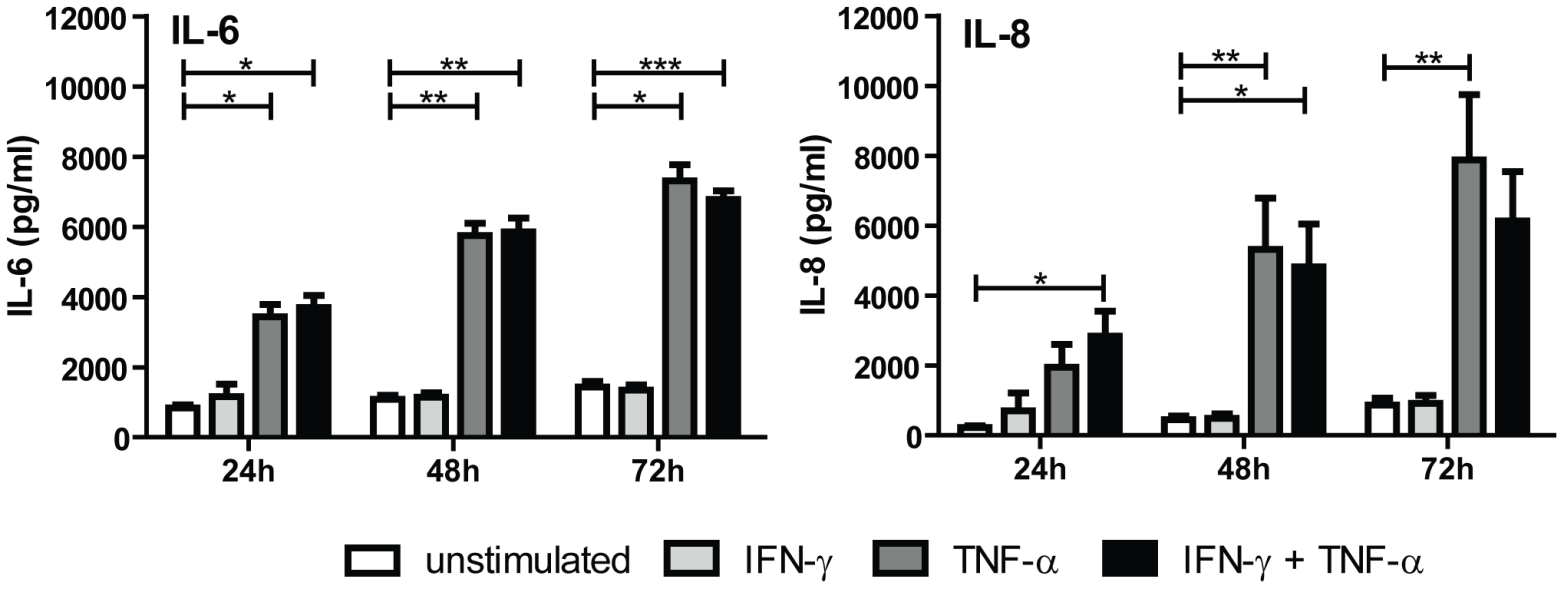
TECs produce proinflammatory cytokines IL-6 and IL-8 after IFN-γ and TNF-α stimulation. Cytokine production was tested using Bio-Plex multiplex assay after TEC stimulation with IFN-γ and TNF-α for 24, 48 and 72 hours (mean ± SEM, n = 10). The proinflammatory cytokines IL-6 and IL-8 were found to be produced by stimulated TECs abundantly. * Significant increase (P <0.05), ** Significant increase (P <0.01).

To define whether TECs are capable of production of cytokines by which naïve CD4^+^ T cells may differentiate into Th1 and/or Th17 cells or change the cytokine profile of Th1 and Th17 cells, we stimulated TECs (N = 10) with IFN-γ/TNF-α and analyzed the cytokine profile of supernatants harvested under these stimulatory conditions compared to the unstimulated state. Unstimulated and IFN-γ/TNF-α activated TECs did not secrete IL-12p70. Th17 associated cytokines including IL-1β, IL-17, IL-23 and TGF-β1 were not produced either by unstimulated TECs or after IFN-γ/TNF-α stimulation (Table 1). In contrast, IL-6 and IL8 were produced in high concentrations by TECs after IFN-γ/TNF-α stimulation compared to unstimulated TECs (5 fold increase, Figure 2).

**Table 1:** Th1 and Th17 differentiation cytokines after 72 hours of stimulation.

Cytokine	Unstimulated TEC	Stimulated TEC
IL-1β	N.D.	N.D.
IL-6	+	+++
IL-12p70	N.D.	N.D.
IL-17	N.D.	N.D.
IL-23	N.D.	N.D.
TGF-β1	N.D.	N.D.

N.D. not detectable; + cytokine present +++ significant upregulation; Stimulated TEC using combination of IFN-γ (50 ng/ml) and TNF-α (20 ng/ml).

### Th1 associated chemokines are produced after IFN-γ and TNF-α stimulation

**Figure 3:**
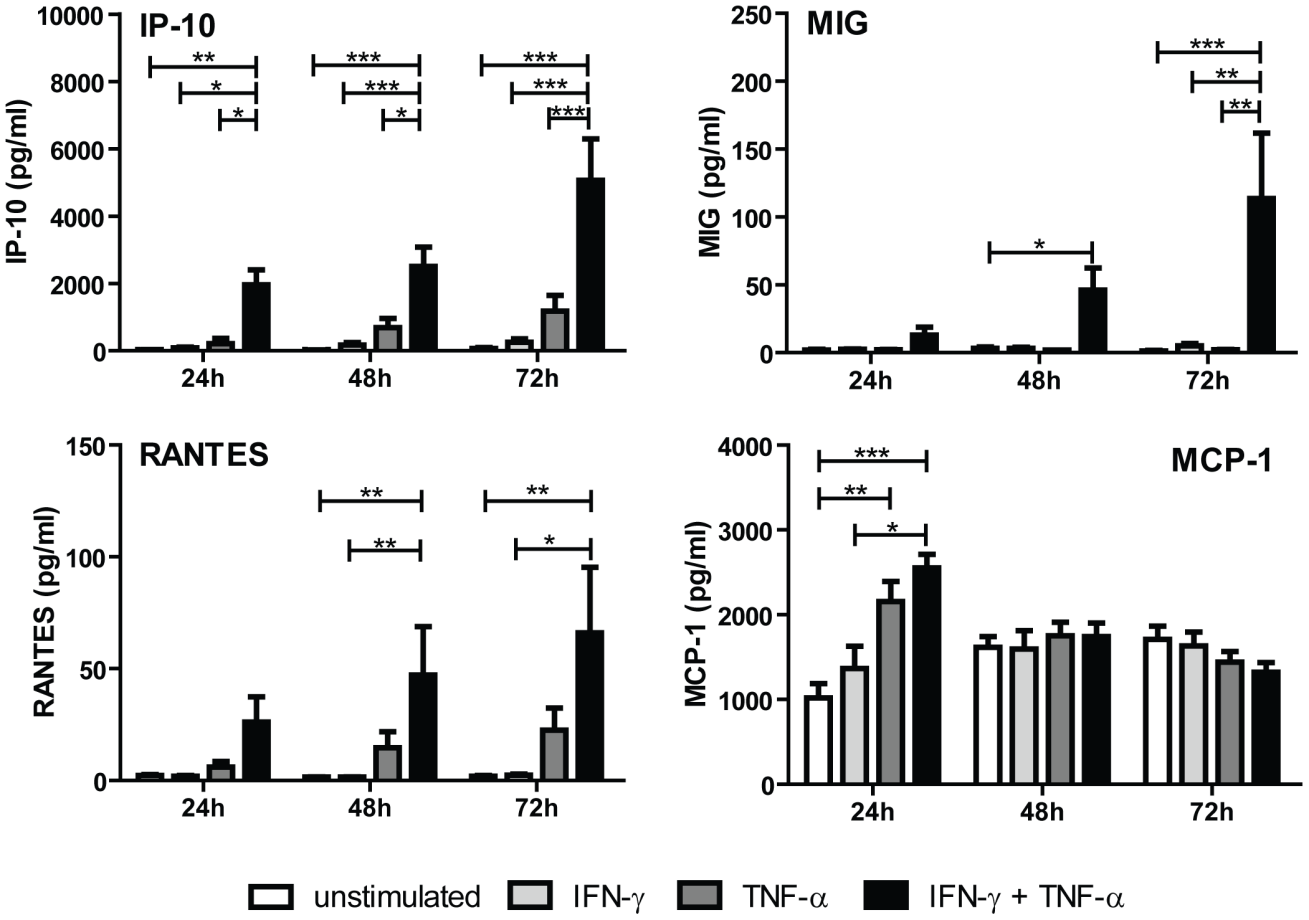
Th1 associated chemokines are produced by TECs after IFN-γ and TNF-α stimulation. TECs were stimulated with IFN-γ (50 ng/ml) and TNF-α (20 ng/ml) for 24, 48 and 72 hours. Supernatant was analyzed for the Th1 associated chemokines CXCL9, CXCL10, CCL2 and CCL5 using Bio-Plex multiplex assay technology. Data shown as mean ± SEM, n = 10. * Significant increase (P <0.05), ** significant increase (P <0.01), *** significant increase (P <0.001).

We investigated the production of Th1 associated chemokines CXCL9 (MIG), CXCL10 (IP-10), CCL5 (RANTES) and CCL2 (MCP-1) under non stimulatory and IFN-γ/TNF-α stimulatory conditions in a time dependent manner for 24, 48 and 72 hours. Combined stimulation with IFN-γ and TNF-α resulted in a synergistic induction of CXCL9, CXCL10 and CCL5. Compared to unstimulated condition CXCL10 showed a 65 fold increase after 24 hours stimulation (30 pg/ml vs 1960 pg/ml; P<0.001). After 72 hours a 2.5 fold increased production of CXL10 was found compared to 24 hours (5064 pg/ml) (Figure 3). CCL5 was already significantly upregulated after 48 hours (47 pg/ml) and remained high after 72 hours (66 pg/ml) as compared to unstimulated condition, while CXCL9 could only be detected after 72 hours stimulation (114 pg/ml). CCL2 production by TECs was present constantly at all time points measured showing a rapid onset and reaching a high plateau level after 24 hours which was retained during 72 hours. In unstimulated condition and at 24 hours, TECs produced CCL2 at a concentration of 1018 pg/ml. IFN-γ alone failed to upregulate CCL2 production, while both TNF-α (2154 pg/ml) and IFN-γ/TNF-α (2550 pg/ml) stimulation significantly upregulated CCL2 production (Figure 3).

### CCL20 is not produced by TECs after IFN-γ and TNF-α stimulation

**Figure 4:**
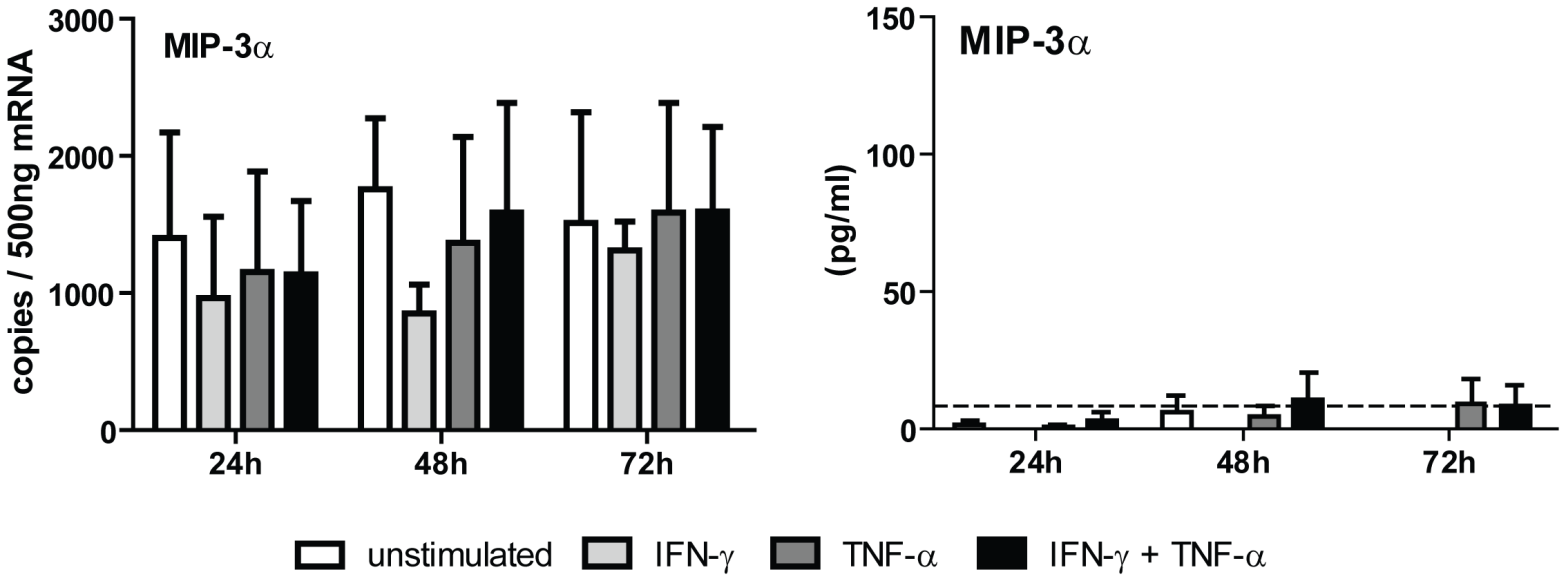
CCL20 is not produced by TECs after IFN-γ and TNF-α stimulation. TECs were IFN-γ/TNF-α stimulated for 24, 48 and 72 hours. CCL20 mRNA expression by TECs was analyzed by PCR and the supernatant was analyzed for CCL20 protein using ELISA. The dotted line represents the lowest CCL20 detection limit (7.8 pg/mL). No significant CCL20 production could be detected after IFN-γ/TNF-α stimulation (n = 8).

We investigated whether tubular epithelial cells have the potential to produce CCL20 (MIP-3α) in our system. TECs did not upregulate CCL20 mRNA levels after IFN-γ and TNF-α stimulation. Accordingly, TECs were also not able to produce the Th17 associated chemokine CCL20 (Figure 4); neither unstimulated nor after 24, 48 and 72 hours of stimulation with IFN-γ and TNF-α.

### Th1 and not Th17 cells are attracted by IFN-γ and TNF-α stimulated TECs

Anti-CD3/CD28 activated PBMCs were added to the upper chamber of 3 µm pore membrane inserts. Unstimulated or IFN-γ/TNF-α stimulated TECs were tested for their capacity to attract CD4^+^CXCR3^+^ or CD4^+^CCR6^+^ T cells, representing respectively Th1 and Th17 containing cell pools (Figure 5A). After 4 hours of incubation, cells in the lower chamber were harvested and CXCR3 and CCR6 expression on CD4^+^ T cells were determined. CD7 positive and CD16/CD56 negative lymphocytes were discriminated as anti-CD3/CD28 activated T lymphocytes (Figure 5B). The total numbers of migrated CD4^+^ T cells (Figure 5C) were measured as readout. Although unstimulated TECs were able to attract CD4^+^CXCR3^+^ T cells to a low extent, IFN-γ/TNF-α activated TECs induced a significant migration of CD4^+^CXCR3^+^ T cells (P<0.05). No CD4^+^CCR6^+^ T cells were attracted by unstimulated TECs. In line with our chemokine data, IFN-γ/TNF-α activated TECs failed to induce migration of CD4^+^CCR6^+^ T cells. As such, activated primary renal tubular epithelial cells attract CD4^+^CXCR3^+^ T cells, but not CD4^+^CCR6^+^ T cells (Figure 5D and Figure 5E).

**Figure:**
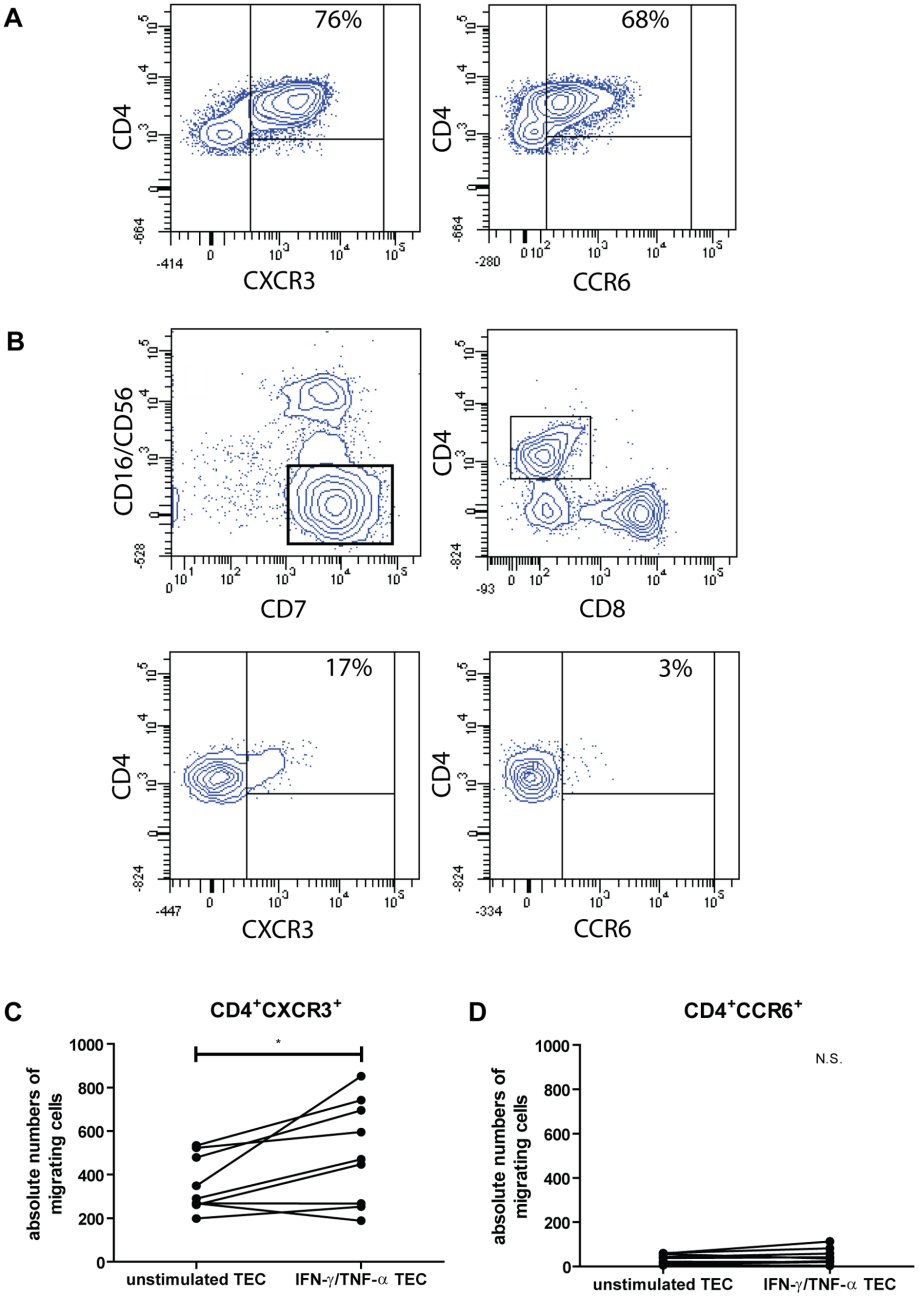
Differential effects of renal tubular epithelial cells on T-cell migration. (A) PBMCs were activated with anti-CD3/CD28, IL-2 and IFN-γ and placed in a 3.0 µm pore transwell system upon unstimulated TECs or 24 hours IFN-γ/TNF-α stimulated TECs. Activated CD4^+^ T cells expressed 76% CXCR3 and 68% CCR6. (B) Significant migration of CD4+CXCR3+ T cells, containing Th1 cell pool, by activated TECs was observed (P<0.05). No migration of CD4+CCR6, containing Th17 cell pool, could be detected. CD7^+^ and CD16^-^/CD56^-^ lymphocytes were discriminated as anti-CD3/CD28 activated T lymphocytes. (C and D) Transwell migration results of 9 individual experiments using unstimulated TECs vs. stimulated TECs are shown. The total cell number of migrated CD4^+^CXCR3^+^ and CD4^+^CCR6^+^ T cells are depicted on the Y –axis. Results were obtained by flow cytometry using fixed time point and fixed volume. * Significant increase (P <0.05).

## Discussion

In this study, we investigated the differential effects of activated human renal epithelial cells on T cell migration. We questioned whether TECs after stimulation by IFN-γ and TNF-α representing instrumental proinflammatory cytokines during rejection, have the potential to attract Th1 and Th17 T-cell subsets. Our data demonstrate that IFN-γ/TNF-α activated renal tubular epithelial cells primarily produce Th1-associated chemokines and not the Th17-associated chemokine CCL20. Functionally, only CD4^+^CXCR3^+^ T cells and hardly any CD4^+^CCR6^+^ T cells were attracted by activated TECs, when PBMC migration towards activated and resting TECs was tested using chemotaxis assays. Moreover, activated TECs do not produce cytokines promoting Th17 cell differentiation in our experimental system.

The recruitment of T cells from the peripheral circulation into the allograft is an essential event in acute kidney transplant rejection. Essential elements involved in this process are chemokines secreted in the tissue and the corresponding chemokine receptors present on T cells [14]. On the one side, transplantation procedure itself may have a direct effect on the chemokine profile within the graft. Following organ harvest, the intervening period of ischemia and subsequent reperfusion results in the release of various pro-inflammatory mediators like IL-1, TNF-α and IL-8 [22]. On the other side, rejection process can lead to release of chemokines which are mainly produced by inflamed parenchymal cells like tubular epithelial cells indicating a crucial role in the attraction of immune cells. It has been demonstrated that in kidney transplant biopsies from patients with acute rejection, expression of CCL2 and CCL5 is mainly found in TECs with mononuclear cell infiltration [23]. The production of chemokines like CCL2, CCL5 and IL-8 *in vitro* by TECs depends on cytokine activation. Various proinflammatory cytokines and CD40L stimulation resulted in a strong production of these chemokines by TECs [24], [25]. Also IL-4 and IL-13 have a dose-dependent effect on CD40-induced CCL5 production by TECs [26]. Activation of TECs *in vitro* is assumed to mimic *in vivo* processes. In our *in vitro* experiments we stimulated the TECs with IFN-γ and TNF-α. This choice was made upon many *in vivo* and *in vitro* observations designating both IFN-γ and TNF-α as key regulator cytokines during kidney transplant rejection. A high TNF-α producer phenotype correlated with recurrent acute rejection episodes clinically [27] and also IFN-γ gene transcription is associated with high grade scores of tubulitis and the occurrence of rejection [28], [29]. Not surprisingly, these proinflammatory cytokines are both upregulated during renal allograft rejection [30].

While Th1 differentiated cells preferentially express CXCR3 and CCR5, Th17 cells preferentially express the chemokine receptor CCR6 [12], [17]. Increased expression of CXCR3 transcript levels were found in both subclinical and clinical acute kidney transplant rejection [31]. It is even suggested that CXCR3 is the most important chemokine receptor for the development of rejection, as CXCR3^-/-^ mice showed an prolonged graft survival compared to wild type mice [29]. Although CXCR3 has been suggested to act as a potent chemokine receptor present during renal allograft rejection, the role of CCR6^+^ T cells still needs to be elucidated. As we were interested in the attraction of Th1 and Th17 cells by TECs, we determined the total number of migrated CD4^+^CXCR3^+^ and CD4^+^CCR6^+^ T cells in our migration assay experiments. TECs were observed to have the potential to attract CD4^+^CXCR3^+^ T cells. Activation of TECs with IFN-γ and TNF-α increased the migration of CD4^+^CXCR3^+^ T cells, while no migration of CD4^+^CCR6^+^ T cells could be detected. Our data confirm that activated TECs are capable of attracting Th1 cells while this is not the case for Th17 cells, assuming that the majority of Th1 cells are CXCR3^+^ T cells and the majority of Th17 cells express CCR6 receptor.

CCR6 expression is of critical importance for Th17 migration and has been associated with aggravated experimental glomerulonephritis, graft-versus-host disease (GVHD), inflammatory bowel disease and rheumatoid arthritis [32]. So far, Woltman et al published the only report on CCL20 expression and CCR6^+^ cells in kidney transplant rejection [19]. The authors reported CCL20 and CCR6^+^ infiltrating cells in kidney graft biopsies during rejection and found that the attraction of immature dendritic cells by TECs depends on CCL20. In our study, the Th17 associated chemokine CCL20 was not produced by TECs after IFN-γ and TNF-α stimulation, while Woltman et al. found an increased CCL20 production after IL-1α and CD40L stimulation [19]. They also reported that after IFN-γ stimulation, a clear inhibitory effect on the IL-1α and CD40L-induced CCL20 production was found. Apparently, CCL20 produced by TECs depends on the type of stimulation.

Chemokines present in the allograft determine the attraction of specific T cell subsets recruited towards this allograft. CXCL9, CXCL10, CCL2 and CCL5 attract Th1 cells [14], while CCL20 attract Th17 cells [17]. We found a strong synergy between the cytokines IFN-γ and TNF-α on chemokine production by TECs. IFN-γ or TNF-α alone was not able to significantly increase CXCL9, CXCL10 or CCL5 at any time point tested, while a combination of IFN-γ and TNF-α increased the chemokine production by TECs profoundly. Synergistic effects of combined stimuli on TEC activation and cytokine/chemokine production have been observed before [24]. In our system, the production of CCL2 was strongly increased when IFN-γ and TNF-α were combined. IFN-γ alone was not able to upregulate CCL2 production while TNF-α could. During kidney transplant rejection intrarenal RNA expression of Th1-associated ligands CXCL10 and CCL5 have been documented [14], also increased urinary chemokines CXCL9 and CXCL10 were found during acute rejection compared to stable recipients [33]. In line, we found Th1-associated chemokines, while we were not able to document the production of the Th17-associated chemokine CCL20 by activated TECs. This indicates that the controversy in literature on CCL20 contribution to rejection probably depends on the dominant cytokines present in the microenvironment.

Local immunoregulation driven by cytokines during kidney graft rejection has been suggested by animal and clinical transplantation studies using graft biopsies and confirmed by *in vitro* studies using TEC cell lines. A variety of proinflammatory cytokines were shown to be produced during rejection like IL-6, TNF-α, IL-15 and IL-18 on both gene transcript and protein level [23]. In our study, proinflammatory cytokines like IL-1β, IL-12p70 and IL-23 were not produced by TECs in contrast to abundantly high IL-6 and IL-8 concentrations. Also active TGF-β1 could not be produced by both resting and activated TECs. As IL-12 is critical for the Th1 response [34] and IL-1β, IL-6, IL-23 and TGF-β1 are critical cytokines for Th17 cell differentiation and development [35], it can be argued whether under our experimental conditions, TEC associated cytokine profile might facilitate the Th1 and Th17 differentiation of naïve CD4^+^ T cells. Dissection of the individual contributions of the CD4^+^ T-cell subsets in transplant rejection is still a challenge for transplant immunologists because of redundancy and plasticity of the different effector pathways of CD4^+^ T cells [36]. Under the influence of IL-12, Th17 cells can switch their phenotype to become Th1 cells, as Th17 express both IL-12 and IL-23 receptors. In contrast, Th1 cells cannot undergo this phenotype switching [37]. The plasticity of the CD4+ T-cell subsets on the one hand and the variety of locally produced inflammatory mediators like cytokines and chemokines *in vivo* on the other hand, challenge the balanced regulation between rejection and acceptance of the kidney graft in due time and make it susceptible for any inflammatory insult which can influence the determinants of this redundant process. We could not find any evidence for a stereotyped cytokine profile by TECs in our system that could direct any phenotype switching and reprogramming of CD4^+^ T cells with regard to Th1- and Th17-associated pathways.

In summary, recruitment and infiltration of a kidney transplant by T cells, is one of the initial steps towards induction of structural graft damage. Our observations show that activated primary renal tubular epithelial cells do not attract Th17 cells nor produce cytokines promoting Th17 cell differentiation.
